# Exciton Absorption and Luminescence in i-Motif DNA

**DOI:** 10.1038/s41598-019-52242-1

**Published:** 2019-11-05

**Authors:** Zakhar V. Reveguk, Evgeny V. Khoroshilov, Andrey. V. Sharkov, Vladimir A. Pomogaev, Andrey A. Buglak, Alexander N. Tarnovsky, Alexei I. Kononov

**Affiliations:** 1 Department of Molecular Biophysics and Polymer Physics, Saint-Petersburg State University, 199034 St. Petersburg, Russia; 2 P.N. Lebedev Physical Institute, Russian Academy of Sciences, 53 Leninsky Pr., 119991 Moscow, Russia; 3 Department of Physics, Tomsk State University, Tomsk, 634050 Russia; 4 Department of Chemistry and Green-Nano Materials Research Center, College of Natural Sciences, Kyungpook National University 1370 Sankyuk-dong, Buk-gu, Daegu 702-701 Republic of Korea; 5 Department of Chemistry and the Center for Photochemical Sciences, Bowling Green State University, Bowling Green, OH USA

**Keywords:** Biological fluorescence, Biological physics, Photochemistry, Physical chemistry

## Abstract

We have studied the excited-state dynamics for the i-motif form of cytosine chains (dC)_10_, using the ultrafast fluorescence up-conversion technique. We have also calculated vertical electronic transition energies and determined the nature of the corresponding excited states in a model tetramer i-motif structure. Quantum chemical calculations of the excitation spectrum of a tetramer i-motif structure predict a significant (0.3 eV) red shift of the lowest-energy transition in the i-motif form relative to its absorption maximum, which agrees with the experimental absorption spectrum. The lowest excitonic state in i-(dC)_10_ is responsible for a 2 ps red-shifted emission at 370 nm observed in the decay-associated spectra obtained on the femtosecond time-scale. This delocalized (excitonic) excited state is likely a precursor to a long-lived excimer state observed in previous studies. Another fast 310 fs component at 330 nm is assigned to a monomer-like locally excited state. Both emissive states form within less than the available time resolution of the instrument (100 fs). This work contributes to the understanding of excited-state dynamics of DNA within the first few picoseconds, which is the most interesting time range with respect to unraveling the photodamage mechanism, including the formation of the most dangerous DNA lesions such as cyclobutane pyrimidine dimers.

## Introduction

It is well known that sun light is a mutagenic agent that causes various DNA damage^1–4^. DNA photoproducts mostly result from direct photochemical reactions in DNA exposed to UVB solar radiation. On the other hand, photochemical reactions can be explored in genome editing^5^. The primary and subsequent photochemical reactions following UV excitation may occur very fast. For example, cyclobutane pyrimidine dimers are shown to form ~1 ps following UV excitation^6,7^, implying no rearrangement of the stacked bases. Answering the questions like what is the character of excited states from which the photochemical reactions start and what is the nature of the photochemical reaction pathway is of vital importance for the understanding of the fundamental principles of DNA photochemistry. These primary photoprocesses occur on a femtosecond time scale and greatly affect the subsequent photochemistry. They have been of intense research interest during the past decade^8–10^. Also, excited-state properties of DNA are of interest to exploit charge transport through DNA with the relevance to both cellular processes and DNA-based devices^11,12^. Owing to the significant progress in laser techniques, ultrafast time-resolved spectroscopy studies became a logical continuation of the earlier steady-state spectroscopy (absorption, luminescence, and circular dichroism (CD)) studies in which the important features of DNA electronic excited states were observed, such as extremely low fluorescence quantum yields and formation of excitons and excimers^13–16^.

The electronic excitation can be harmful to DNA, and nature has created effective, but non-ideal deactivation mechanisms to minimize its destructive consequences. In single bases, electronic curve crossings (conical intersections) have been proposed to lead to the fast radiationless deactivation of the singlet excited state within the first picosecond^8,9^. Yet, even for the monomers, there exists different interpretations of the nature of the relaxation path and reaction intermediates^17^. The situation is much more complicated in DNA, where nucleobases interact through stacking and base pairing. The Franck-Condon excited state of DNA is mostly of an excitonic nature, which has been understood since the 1960’s thanks to works of Tinoco, Cantor, Bush and others who studied CD spectra of DNA^14,18,19^. Since then, many theoretical works using various levels of theory, from semi-empirical to high-level ab-initio methods, gave evidence for excitonic interactions, which is reviewed, for example, in ref.^20^. It should be noted that these theoretical approaches deal with Franck-Condon excited states. However, the question is how long the exciton state lasts in DNA. In this respect, so far there is no conclusive experimental evidence that the delocalized states persist even on a femtosecond time scale, which would not be surprising taking into account small values of exciton splitting (~0.1 eV)^21^ in comparison to the DNA absorption band width (~0.5 eV). Homopolynucleotides are widely used models in studying the excited-state dynamics in the stacked nucleobases. However, even for the most well-studied polyadenilyc DNA strands, the conclusions made regarding the primary photoprocesses have often been controversial. For example, Kohler *et al*. believed that long-lived charge-transfer states (excimers) are formed directly from the Franck-Condon state of the stacked bases^22,23^. Phillips *et al*. discussed the dynamics of the excited states already localized on a single individual base^24^. As was noted in refs^23,25^, exciton localization in DNA most likely occurs faster than experimental time resolution (<100 fs). Based on quantum chemical calculations, Improta and Barone showed that the excited state dynamics in stacked adenines is complex, including localization of the excitation on a single base, “neutral” and “charge-transfer”excimers formation, involving a decrease of the stacking distance^26^. This complex picture agrees with a complex multiexponential decay of the excited states observed in polyadenines^24,27^. Based on UV/IR pump–probe experiments, Fiebig *et al*. suggested that an exciton state delocalized over three-to-four bases in polyadenines is present on a picosecond time scale^28^. However, Kohler *et al*. have refuted that result and explained it as being due to base-stacking disorder^23^. Markovitsi *et al*. believed that the lower initial fluorescence anisotropy value and its faster decay in the polymer compared to monomers suggested that a part of the DNA fluorescence stems from the exciton states^27,29–32^. On the other hand, these features of the anisotropy might be explained by the complex dynamics involving both rapid (faster than time resolution) localization of the excitation and subsequent excimers formation^26^.

In this work, using the ultrafast fluorescence up-conversion technique, we study the excited state dynamics in cytosine DNA tracts. Specifically, we have studied the excited-state dynamics for both neutral and hemi-protonated cytosine chains (dC)_10_. The latter assembles into a so-called i-motif structure. The i-motif structure is of interest in biology^33^ as well as in nanotechnology applications^11,34,35^. Direct excitation of cytosines is also known to lead to formation of mutagenic photoproducts that are not efficiently repaired in cells^36,37^, so studying stacked cytosines is important for establishing the correlation between the initial conformation, nature of excited state and photodamage. It is also worth noting that the absorption spectrum of i-motif structures is substantially red-shifted, where the intensity of solar UVB light is on an upward trend with the wavelength, which makes i-motif one of the preferred targets of solar radiation.

Whereas monomeric cytosine derivatives were extensively studied^38–44^, only a few studies have dealt with the excited-state dynamics in cytosine homopolynucleotides on a picosecond time scale^45–48^. In particular, Kohler^46^ and Quinn^48,49^ with co-authors found that UV excitation in i-motif structures resulted in the formation of a significant fraction of long-lived (hundreds of ps) excited states, which could be attributed to charge-transfer excimers. Plessow *et al*. observed long-lived (ca. 100 ps and 2 ns) fluorescence components at 400 nm in (dC)_15_^45^, assigned by Kohler *et al*.^46^ to the hemi-protonated structure present at neutral pH.

In the present study, we found that the hemi-protonated i-motif structure of (dC)_10_ exhibits two spectrally different components due to the local (monomer-like) and delocalized (exciton) emissive states both formed from the Franck-Condon excited state in less than 100 fs.

## Methods and Materials

### Experimental details

The HPLC purified (dC)_10_ was purchased from Syntol (Moscow, Russia) and deoxycytidine-5’-monophosphate (dCp) was acquired from Sigma-Aldrich. In a typical sample preparation, DNA strands or monomers were dissolved in water (pH 6), mixed with the proper buffer, borate (pH 9) or citrate (pH 3), to maintain the required pH. For all samples, optical density at 266 nm was equal to 1 (a 4-mm pathlength quartz cuvette) and to 1.5 (a 0.4 mm pathlength rotating silica cell) in steady-state and time-resolved fluorescence measurements, respectively.

Absorption spectra were acquired using a SPECORD^®^ 210 PLUS (Analytik Jena) spectrophotometer. CD spectra were measured using a J-815 Circular Dichroism spectrometer (Jasco). Absorption and CD measurements were carried out in 4 mm path length quartz cuvettes.

Steady-state fluorescence measurements spectra were measured using a F-6000 (Shimadzu) fluorimeter. The solutions were kept in a 4-mm path length quartz cuvette at room temperature. The emission spectra were corrected for the instrument spectral sensitivity.

The fluorescence time-resolved measurements were carried out using a FOG 100-DX fluorescence up-conversion spectrometer (CDP Corp., Moscow, Russia). Excitation light pulses were provided by the third harmonic (266 nm) of a mode-locked Ti: sapphire laser (TISSA, CDP Corp.) operating at 80 MHz repetition rate. The 3-mm diameter 8-mW UV output beam was focused by a 100-mm lens into the sample solution kept in a 0.4-mm path length rotating silica cell. The excitation power was about 10 mW and the excitation spot diameter was about 200 µm. All measurements were performed at room temperature under aerated conditions. The width of the instrument response function was evaluated to be 400 ± 50 fs (FWHM, Gaussian-shaped), as determined by the fits of the luminescence decay curves of tryptophan and Coumarin 30 solutions as well as by the signal from neat water due to Raman scattering. The polarization of the excitation light was controlled by a Berek compensator. Parallel (*I*_*par*_) and perpendicular (*I*_*perp*_) kinetic traces were recorded by controlling the polarization of the excitation beam with a Berek compensator. The total fluorescence kinetic traces were recorded at magic angle or obtained by calculating the following quantity: *I*_*par*_ + *2 I*_*perp*_.

### Quantum chemical calculations

**Figure 1:**
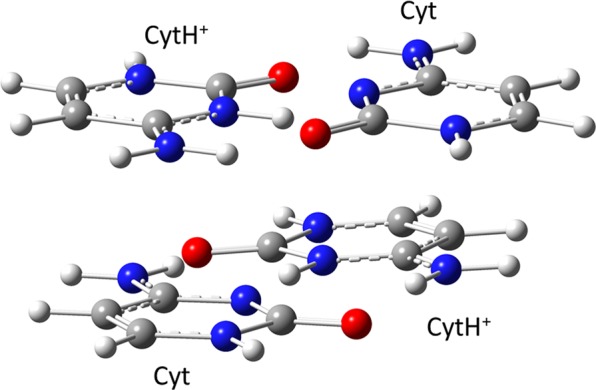
Structure of the i-motif tetramer used in our calculations.

The geometry of the semi-protonated cytosine tetramer (Cyt_4_2H^+^, shown in Fig. 1) was constructed by first optimizing the monomers and then inserting them into an arrangement of the initial tetramer geometry in i-motif (PDB: 1ELN^50^). The geometries of neutral (Cyt) and protonated at N3 atom (CytH^+^) monomers were optimized using the Møller−Plesset second-order perturbation theory (RI-MP2)^51^ with the aug-cc-pVDZ^52^ basis set as implemented in Orca v.3.0 program package^53^. The electronic excitation spectra of the molecular systems were calculated in Turbomole v.7.2 program package^54^ using the second-order algebraic-diagrammatic construction ADC(2) method^55^ utilizing the hybrid (cc-pVDZ for hydrogen and aug-cc-pVDZ for other elements) basis sets, and both full and frozen occupied MOs^56^. The implicit COSMO solvent model was used. The frozen core approximation method decreased computational time significantly without loss of accuracy. Earlier, the same approach was found to be appropriate for calculations of the nucleobase dimer spectra^57^. Methyl substitution is often used to mimic an electron-donating effect of the ribose group. We found an acceptable agreement between cytosine and 1-methylcytosine in their excited state energies **(**Table S1**)**, which allowed us to use cytosine as a monomer unit instead of 1-methylcytosine when calculating the excitation spectra of the i-motif tetramer.

## Results and Discussion

**Figure 2:**
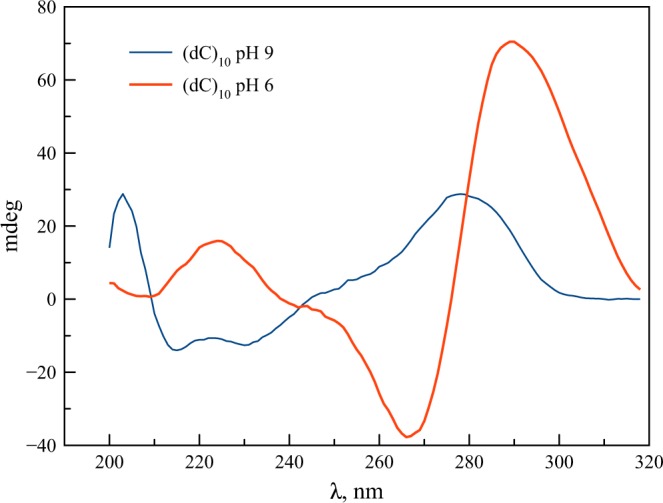
CD spectra of (dC)_10_ at pH 6 and 9.

In water (pH 6), (dC)_10_ is in a hemi-protonated conformation with C^+^-H^…^C pairing, as can be seen from the CD spectrum (Fig. 2) typical for hemi-protonated cytosine tracts^58,59^. This results in the formation of the so-called i-motif (i-(dC)_10_)^60^. Hemi-protonated cytosine tracts exhibit long-lived excimer states^46,48^. At pH 9, (dC)_10_ is in the neutral single-stranded conformation, ss-(dC)_10_, Fig. 2. The pKa value for the oligonucleotide increases to 7 in comparison with the monomer pKa 4.2^34^. The UV titration of (dC)_10_ gives the same pKa 7 (Fig. S1).

**Figure 3:**
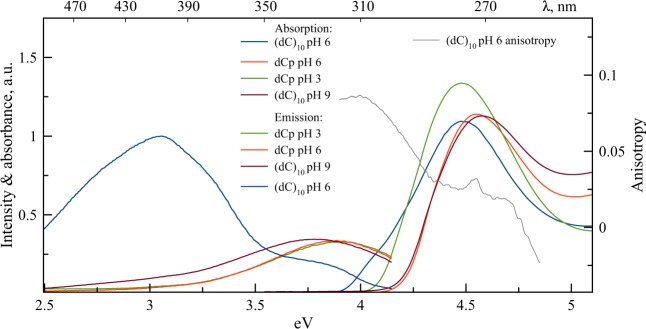
Absorption, fluorescence (λ_ex_ = 260 nm), and fluorescence excitation anisotropy (λ_em_ = 400 nm) spectra of dCp and (dC)_10_ at different pH values. The absorbance values are given per base, with the hypochromic effect being determined from the melting curve of (dC)_10_ at pH 9. The fluorescence emission spectra are λ^2^-scaled.

The absorption and fluorescence steady-state spectra of dCp and two forms of (dC)_10_ are presented in Fig. 3. While the absorption spectrum of the single-stranded conformation of (dC)_10_ is very close to the spectrum of monomer dCp at neutral pH, the absorption spectrum of the hemi-protonated i-motif structure differs from both the neutral and protonated (pH 3) dCp spectra. The spectrum also exhibits a shoulder around 4 eV, indicating a low-energy electronic transition. A complex structure of the overall absorption band is also revealed by the fluorescence excitation anisotropy curve (Fig. 3), indicating the presence of at least two electronic transitions within the UV band. As compared with the ss-(dC)_10_, such behavior of the i-(dC)_10_ absorption spectrum is evidently the manifestation of strong exciton interactions. The conservative CD spectrum of i-(dC)_10_ is also a consequence of the exciton interaction. The exciton splitting in the absorption spectrum of the i-motif structure can approximately be estimated as ca. 0.4 eV (the difference between the positions of maximum and shoulder in the spectrum), which is several times greater than the typical values in DNA^21^. In principle, exciton as well as charge-resonance and charge-transfer terms can contribute to the low-energy electronic transition at the long-wavelength tail of the spectrum. A detailed description of the structure of the excited states in (dC)_10_ requires advanced QM calculations.

**Figure 4:**
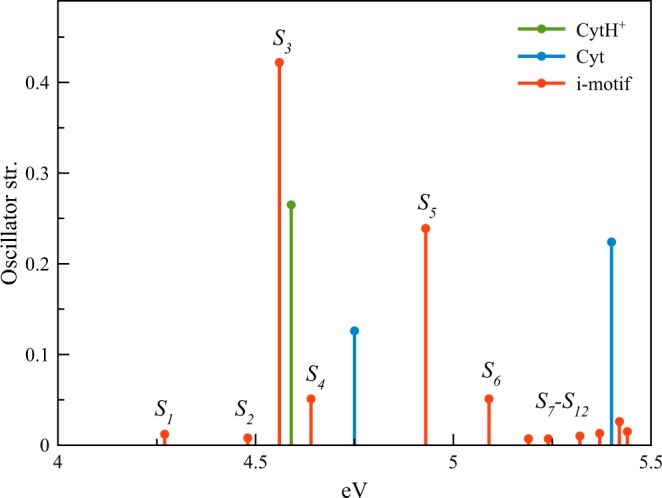
Calculated stick spectra of the protonated and neutral cytosine monomers and the cytosine tetramer derived from the PDB-based i-motif structure.

We have calculated the absorption spectrum of the semi-protonated tetramer derived from the crystal i-motif 1ELN structure taken from the PDB database^50^ (Fig. 1). The stick spectra of the protonated and neutral monomers and semi-protonated i-motif species are shown in Fig. 4 and Table S2. The calculated spectra of the i-motif and neutral and protonated monomers qualitatively reproduce the relative maximum positions and intensities in the experimental absorption spectra shown in Fig. 3. Overall, the calculated maxima are blue-shifted by 0.1 eV relative to the experimental maxima. A more quantitative agreement with the experimental spectra could probably be reached if the explicit water environment was included. Additional work, including thorough consideration of the effects of DNA dynamics and environment, is needed. It requires more complicated QM/MM studies.

The protonated and neutral monomers have the lowest S_1_ (π→π*) transitions at 4.59 and 4.75 eV respectively, which splits into π→π* states in the tetramer. The charge difference densities (CDD) plotted in Table S2 show that these states are delocalized over two-to-four monomer units and have excitonic character with some admixture of charge transfer (CT) or charge resonance (CR) states. More detailed analysis requires additional calculations. It is important, that in the i-motif, the lowest π→π* transition is red-shifted by ca. 0.3 eV from the maximum due to a significant exciton interaction. For comparison, typical values of the exciton splitting in the B-form of DNA is less than 0.1 eV^21,57^. The large value of this shift calculated for the i-motif agrees with that observed in the experimental absorption spectrum of i-(dC)_10_. The latter exhibits a pronounced low-energy transition(s) at about 4.1 eV (Fig. 3). The red-shifted transitions in some non-canonical stacking forms were predicted using the QM calculations^57^. It has been shown in the same work that the excitonic term is dominant in the lowest-energy electronic states of the base stacking forms. Normally, these are not seen in the absorption spectra of nucleic acids, but in some cases can be observed in the fluorescence excitation spectra^61,62^. i-motif, in this regard, appears to be a unique DNA structure with the strongly red-shifted, low-lying electronic excited state, which is seen in the absorption spectrum.

**Figure 5:**
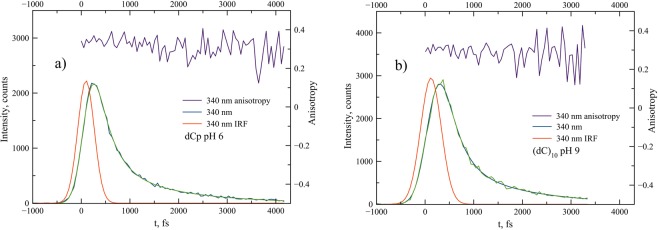
Fluorescence intensity and fluorescence anisotropy decay curves of dCp in water (pH 6) (**a**) and (dC)_10_ at pH 9 (**b**).

**Figure 6:**
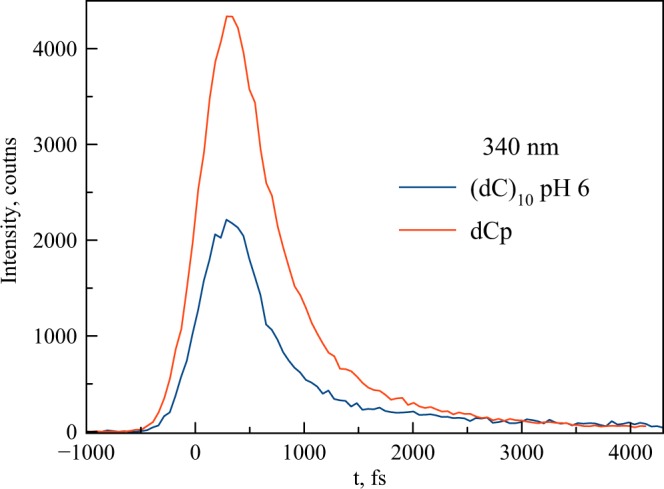
Comparison of the 340-nm fluorescence intensity decay curves of (dC)_10_ in water (pH 6) and hemi-protonated dCp (obtained as the average curve for the neutral and protonated forms). Signals were recorded in back-to-back measurements on solutions having equal absorbance at the exciation wavelength.

**Figure 7:**
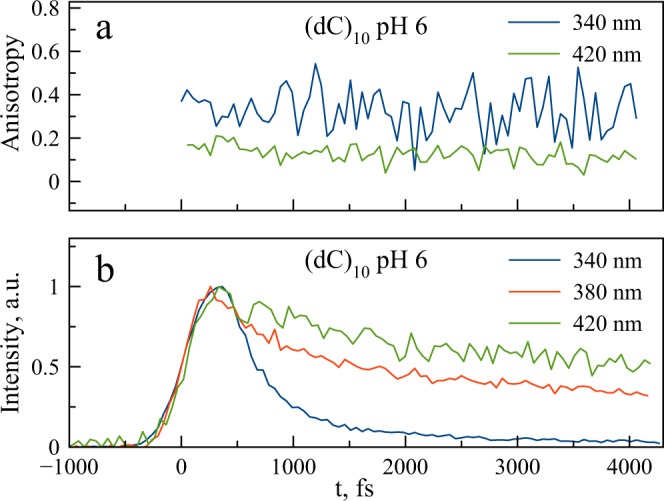
Fluorescence anisotropy (**a**) and intensity (**b**) decay traces of (dC)_10_ in water (pH 6) at different emission wavelengths.

The steady-state fluorescence spectrum of i-(dC)_10_ in comparison with both the monomer forms and ss-(dC)_10_ exhibits a broad long-wavelength emission band at about 400 nm, in addition to the monomer-like emission band at ca. 330 nm (Fig. 3). Both fluorescence and anisotropy decay curves of dCp at neutral pH (Fig. 5a), dCp at pH 3 (Fig. S3), ss-(dC)_10_ at pH 9 (Fig. 5b) and semi-protonated i-(dC)_10_ at pH 6 (Figs 5–7) recorded at 340 nm are practically the same. The results of exponential fits are summarized in Table 1. This means that the nature of the short-wavelength band at 330 nm in the steady-spectra of the polymer and monomer is the same. This emission in the case of the polymer thus can be viewed as “monomer-like” fluorescence from the locally excited state. However, the amplitude in the i-(dC)_10_ decay curve appears to be 2 times less compared to that of the monomer (Fig. 6). This indicates that the yield of “monomer-like” fluorescence in i-(dC)_10_ is about 50%. This cannot be attributed to a hypochromic effect, as the integral absorption of the semi-protonated (dC)_10_ is only ca. 5% less than the averaged absorption of the protonated and neutral monomer (Fig. 3). It is also should be noted that a certain amount of the fluorescence at 340 nm might originate from the single-stranded form present in solution in these experimental conditions^59^. Where does the rest of the excited i-(dC)_10_ go from the Franck-Condon state?

**Table 1:** Decay components of (dC)_10_ and dCp samples (pH 3, pH 6, and pH 9).

Sample	*a*_1_	*t*_1_, fs	*t*_2_, fs	*t*_3_, fs
dCp at 340 nm, pH 6	0.79 ± 0.03	310 ± 30	1400 ± 100	—
dCp at 340 nm, pH 3	0.83 ± 0.07	310 ± 30	800 ± 100	—
(dC)_10_ at 340 nm, pH 9	0.80 ± 0.03	300 ± 30	1400 ± 200	—
(dC)_10_, pH 6 (Global fit)		310 ± 30	2000 ± 200	25000 ± 5000

The experimental data were fitted with a bi-exponential function *a*_1_*exp*(-*t*/*t*_1_) + (1-*a*_1_)*exp*(-*t*/*t*_2_) or with a three-exponential function in the case of global fitting on a longer time scale.

**Figure 8:**
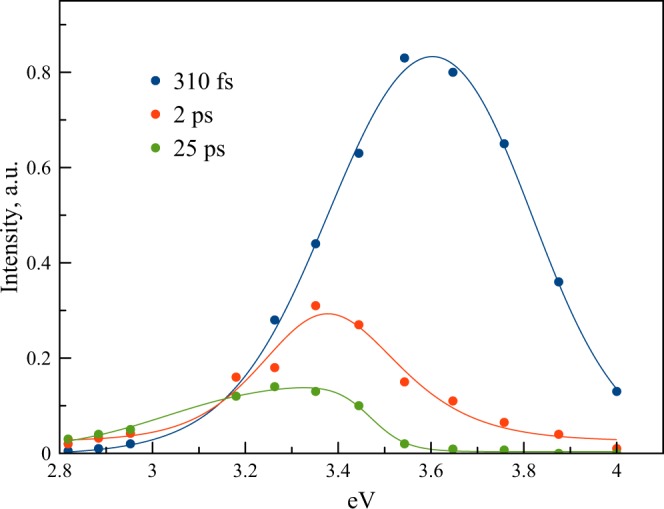
The decay-associated spectra of the slow and fast components for (dC)_10_ in water (pH 6).

We recorded fluorescence decay curves of dCp and (dC)_10_ samples in the range of 310–450 nm. For dCp and ss-(dC)_10_, the decay curves practically do not depend on the wavelength (Figs S2–S4). The decay curves of i-(dC)_10_ recorded at 340, 380 and 420 nm wavelengths are presented in Fig. 7. Other curves are shown in Fig. S5. In the long-wavelength part of the spectrum, the decay curves exhibit slow components that become dominant at 380 nm (Fig. 7). It should be noted that no changes in the decay curves were observed after repeated scan (Fig. S6), which indicated no degradation of the samples. All the decay curves in the range 310–450 nm can be satisfactory fitted with three exponents. The global fit gives the decay times 310 fs, 2 ps, and 25 ps. The decay-associated spectra of the components are shown in Fig. 8.

The short-wavelength component with the decay time of 310 fs is evidently associated with the monomer emission, which has similar decay time and fluorescence anisotropy (although the short-wavelength maximum in the up-conversion spectrum is seemingly red-shifted slightly from the corresponding maximum at about 330 nm in the steady-state spectrum, this shift is caused by the sharp decrease in the set-up sensitivity at the short-wavelength edge of the spectrum). The component with 2 ps decay time (Fig. 8) is located in the long-wavelength part of the spectrum at about 370 nm. The other long-lived component is even more shifted to the red. The fluorescence anisotropy of slow components is significantly less than that of the monomer emission at 340 nm (Fig. 7), suggesting different emission dipole moments.

The red-shifted fluorescence bands in DNAs are commonly assigned to the so-called excimer fluorescence of DNA components^15,16,24^. A high quantum yield of formation of long-lived excimers in hemi-protonated cytosine strands in i-motif conformations has been observed in time-resolved transient absorption measurements^46,48^, but their life time is much longer (hundreds of ps). Some of them are fluorescent^45^ and are seen as the 400 nm emission band in the steady-state spectrum. It should be noted that the term ‘excimer’ (excited dimer) implies two molecules of the same kind, one in its electronic excited state and the other in the ground state, which, when they emerge at a short separation distance, form an excimer as a result of mutual attraction^63^. Theoretical calculations^26,64^ in agreement with experimental observations^23,24^ clearly demonstrate the existence of such states, for example, in the adenine strands with a “face-to-face” base stacking arrangement. Formation of excimers in the case of stacked cytosines is also predicted by the theory^65^ and observed experimentally^45,46,48,49^. A similar red-shifted 360 nm fluorescence band observed for polyadenines was assigned to excimers^24,26^. In our case, there are two factors that argue against the assignment of the 370 nm emission in i-(dC)_10_ to the excimer. First, the rise time of the red-shifted fluorescence in the range 370–450 nm is faster than the available time resolution of the instrument, i.e. less than 100 fs (Figs 7, S5). Also, the significant decrease in the up-conversion signal of i-(dC)_10_ at 340 nm compared with that of dCp (Fig. 6) suggests that the 370 nm emitting state forms directly from the Franck-Condon state rather than from the local monomer excited state. The sub-100 fs time interval is too short for any mutual approach of the bases to occur. Second, the monomer Stokes shift as well as the spectral shift between the lowest-energy ∼4.1 eV transition in the i-(dC)_10_ absorption spectrum (Fig. 3) and the slow component spectral maximum in the fluorescence decay-associated spectrum (Fig. 8) all appear to be the same, ca. 0.8 eV. Thus, the 370 nm emission originates directly from the lowest-energy state in the stacking structure of semi-protonated (dC)_10_ without any structural rearrangement of the bases. The plot of the difference densities (Table S2) suggests that both exciton and CT/CR interactions contribute to the lowest S1 state of the i-motif. In fact, this means that the 370 nm emissive state is a delocalized emissive state.

**Scheme 1:**
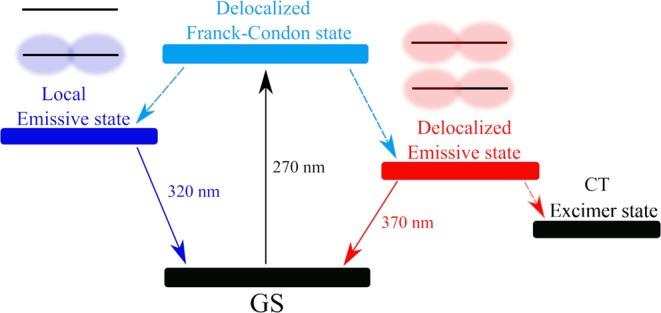
Photoprocesses in i-(dC)_10_.

As the fluorescence life time of the local excited state is not changed in (dC)_10_, it is reasonable to suggest that subsequent charge transfer in the emissive state likely leads to formation of long-lived CT excimers observed on a sub-nanosecond time scale^46,48^. For example, in the case of adenine strands it was suggested very recently that the charge-transfer states form within 3 ps^66^. The proposed scheme of the photoprocesses in the i-(dC)_10_ structure is shown in Scheme 1.

The question arises why the delocalized excitonic state has a relatively long life-time of several picoseconds, which is not observed in other DNA structures. Two factors in the i-(dC)_10_ structure may be responsible for this effect: large value of exciton splitting comparable with the absorption band width and/or contribution of CT/CR term to the lowest-energy exciton state. The more detailed description of the structure and dynamics of the excited states in i-motif requires further theoretical and experimental studies.

## Conclusion

In conclusion, we have studied the nature and dynamics of the excited states for the single-stranded and i-motif forms of cytosine chains (dC)_10_. Quantum chemical calculations of the excitation spectrum of a tetramer i-motif structure predict a significant (0.3 eV) red shift of the lowest-energy transition in the i-motif form relative to its absorption maximum, which agrees with the experimental absorption spectrum. The lowest excitonic state in i-(dC)_10_ is responsible for the 2 ps red-shifted emission at 370 nm observed in the decay-associated spectra obtained on the femtosecond time-scale. Another fast 310 fs component at 330 nm is assigned to a monomer-like locally excited state. The delocalized emissive state is most likely a precursor for the formation of long-lived charge-transfer excimer states observed in i-motif structures.

## Supplementary information

Supplementary figures and tables

## References

[1] Pfeifer GP, You Y-H, Besaratinia A (2005). Mutations induced by ultraviolet light. Mutat. Res. Mol. Mech. Mutagen..

[2] Brash DE (1991). A role for sunlight in skin cancer: UV-induced p53 mutations in squamous cell carcinoma. Proc. Natl. Acad. Sci..

[3] de Gruijl FR (1999). Skin cancer and solar UV radiation. Eur. J. Cancer.

[4] Besaratinia A (2011). Wavelength dependence of ultraviolet radiation-induced DNA damage as determined by laser irradiation suggests that cyclobutane pyrimidine dimers are the principal DNA lesions produced by terrestrial sunlight. FASEB J..

[5] Sethi S, Nakamura S, Fujimoto K (2018). Study of Photochemical Cytosine to Uracil Transition via Ultrafast Photo-Cross-Linking Using Vinylcarbazole Derivatives in Duplex DNA. Molecules.

[6] Schreier WJ (2007). Thymine Dimerization in DNA Is an Ultrafast Photoreaction. Science.

[7] Schreier WJ (2009). Thymine Dimerization in DNA Model Systems: Cyclobutane Photolesion Is Predominantly Formed via the Singlet Channel. J. Am. Chem. Soc..

[8] Middleton CT (2009). DNA Excited-State Dynamics: From Single Bases to the Double Helix. Annu. Rev. Phys. Chem..

[9] Pollum, M., Martínez-Fernández, L. & Crespo-Hernández, C. E. Photochemistry of Nucleic Acid Bases and Their Thio- and Aza-Analogues in Solution. in *Photoinduced Phenomena in Nucleic Acids I* (eds Barbatti, M., Borin, A. C. & Ullrich, S.) **355**, 245–327 (Springer International Publishing, 2014). 10.1007/128_2014_554 25238718

[10] Nielsen LM, Hoffmann SV, Nielsen SB (2013). Electronic coupling between photo-excited stacked bases in DNA and RNA strands with emphasis on the bright states initially populated. Photochem. Photobiol. Sci..

[11] Fujii T (2017). Structure and Dynamics of Electron Injection and Charge Recombination in i-Motif DNA Conjugates. J. Phys. Chem. B.

[12] Thazhathveetil AK, Harris MA, Young RM, Wasielewski MR, Lewis FD (2017). Efficient Charge Transport via DNA G-Quadruplexes. J. Am. Chem. Soc..

[13] Gueron M, Shulman RG (1968). Energy Transfer in Polynucleotides. Annu. Rev. Biochem..

[14] Bush, C. A. Uv spectroscopy circular dichroism and optical rotatory dispersion. *Basic Principles in**Nucleic Acid Chemistry* **91** (1974).

[15] Gueron, M., Eisinger, J. & Lamola, A. A. Excited states of nucleic acids. *Basic Principles in**Nucleic Acid Chemistry* **311** (1974).

[16] Callis PR (1983). Electronic States and Luminescence of Nucleic Acid Systems. Annu. Rev. Phys. Chem..

[17] Prokhorenko VI, Picchiotti A, Pola M, Dijkstra AG, Miller RJD (2016). New Insights into the Photophysics of DNA Nucleobases. J. Phys. Chem. Lett..

[18] Cantor CR, Tinoco I (1965). Absorption and Optical Rotatory Dispersion of Seven Trinucleoside Diphosphates. J. Mol. Biol..

[19] Cantor, C. R. & Shimel, P. R. *Biophysical Chemistry part II*. (W. H. Freeman and Company, 1980).

[20] Improta R, Santoro F, Blancafort L (2016). Quantum Mechanical Studies on the Photophysics and the Photochemistry of Nucleic Acids and Nucleobases. Chem. Rev..

[21] Nachtigallová D, Hobza P, Ritze H-H (2008). Electronic splitting in the excited states of DNA base homodimers and -trimers: an evaluation of short-range and Coulombic interactions. Phys. Chem. Chem. Phys..

[22] Crespo-Hernández CE, Cohen B, Kohler B (2005). Base stacking controls excited-state dynamics in A·T DNA. Nature.

[23] Su C, Middleton CT, Kohler B (2012). Base-Stacking Disorder and Excited-State Dynamics in Single-Stranded Adenine Homo-oligonucleotides. J. Phys. Chem. B.

[24] Kwok W-M, Ma C, Phillips DL (2006). Femtosecond Time- and Wavelength-Resolved Fluorescence and Absorption Spectroscopic Study of the Excited States of Adenosine and an Adenine Oligomer. J. Am. Chem. Soc..

[25] Volkov IL, Reveguk ZV, Serdobintsev PY, Ramazanov RR, Kononov AI (2018). DNA as UV light–harvesting antenna. Nucleic Acids Res..

[26] Improta R, Barone V (2011). Interplay between “Neutral” and “Charge-Transfer” Excimers Rules the Excited State Decay in Adenine-Rich Polynucleotides. Angew. Chem. Int. Ed..

[27] Banyasz A (2013). Multi-Pathway Excited State Relaxation of Adenine Oligomers in Aqueous Solution: A Joint Theoretical and Experimental Study. Chem. - Eur. J..

[28] Buchvarov I, Wang Q, Raytchev M, Trifonov A, Fiebig T (2007). Electronic energy delocalization and dissipation in single- and double-stranded DNA. Proc. Natl. Acad. Sci. USA.

[29] Markovitsi D, Gustavsson T, Talbot F (2007). Excited states and energy transfer among DNA bases in double helices. Photochem. Photobiol. Sci..

[30] Markovitsi D (2006). Complexity of excited-state dynamics in DNA: Molecular spectroscopy. Nature.

[31] Miannay F-A, Bányász Á, Gustavsson T, Markovitsi D (2007). Ultrafast Excited-State Deactivation and Energy Transfer in Guanine−Cytosine DNA Double Helices. J. Am. Chem. Soc..

[32] Vayá I, Gustavsson T, Douki T, Berlin Y, Markovitsi D (2012). Electronic Excitation Energy Transfer between Nucleobases of Natural DNA. J. Am. Chem. Soc..

[33] Zeraati M (2018). I-motif DNA structures are formed in the nuclei of human cells. Nat. Chem..

[34] Zikich D, Liu K, Sagiv L, Porath D, Kotlyar A (2011). I-Motif Nanospheres: Unusual Self-Assembly of Long Cytosine Strands. Small.

[35] Dong Y, Yang Z, Liu D (2014). DNA Nanotechnology Based on i-Motif Structures. Acc. Chem. Res..

[36] Ravanat J-L, Douki T, Cadet J (2001). Direct and indirect effects of UV radiation on DNA and its components. J. Photochem. Photobiol. B.

[37] Douki T (2006). Effect of denaturation on the photochemistry of pyrimidine bases in isolated DNA. J. Photochem. Photobiol. B.

[38] Keane PM (2011). A Comparative Picosecond Transient Infrared Study of 1-Methylcytosine and 5′-dCMP That Sheds Further Light on the Excited States of Cytosine Derivatives. J. Am. Chem. Soc..

[39] Ho J-W (2011). Disentangling Intrinsic Ultrafast Excited-State Dynamics of Cytosine Tautomers. J. Phys. Chem. A.

[40] Kosma K, Schröter C, Samoylova E, Hertel IV, Schultz T (2009). Excited-State Dynamics of Cytosine Tautomers. J. Am. Chem. Soc..

[41] Ho J-W (2015). Microhydration Effects on the Ultrafast Photodynamics of Cytosine: Evidences for a Possible Hydration-Site Dependence. Angew. Chem. Int. Ed..

[42] Blancafort L, Cohen B, Hare PM, Kohler B, Robb MA (2005). Singlet Excited-State Dynamics of 5-Fluorocytosine and Cytosine: An Experimental and Computational Study. J. Phys. Chem. A.

[43] Malone RJ, Miller AM, Kohler B (2007). Singlet Excited-state Lifetimes of Cytosine Derivatives Measured by Femtosecond Transient Absorption¶. Photochem. Photobiol..

[44] Blaser S (2016). Gas-Phase Cytosine and Cytosine-N _1_ -Derivatives Have 0.1–1 ns Lifetimes Near the S _1_ State Minimum. J. Phys. Chem. Lett..

[45] Plessow R, Brockhinke A, Eimer W, Kohse-Höinghaus K (2000). Intrinsic Time- and Wavelength-Resolved Fluorescence of Oligonucleotides: A Systematic Investigation Using a Novel Picosecond Laser Approach. J. Phys. Chem. B.

[46] Cohen B, Larson MH, Kohler B (2008). Ultrafast excited-state dynamics of RNA and DNA C tracts. Chem. Phys..

[47] Schwalb NK, Temps F (2008). Base Sequence and Higher-Order Structure Induce the Complex Excited-State Dynamics in DNA. Science.

[48] Keane PM (2014). Long-lived excited states in i-motif DNA studied by picosecond time-resolved IR spectroscopy. Chem Commun.

[49] Keane PM (2016). Long-Lived Excited-State Dynamics of i-Motif Structures Probed by Time-Resolved Infrared Spectroscopy. ChemPhysChem.

[50] Phan AT, Guéron M, Leroy J-L (2000). The solution structure and internal motions of a fragment of the cytidine-rich strand of the human telomere 1 1Edited by I. Tinoco. J. Mol. Biol..

[51] Bernholdt DE, Harrison RJ (1996). Large-scale correlated electronic structure calculations: the RI-MP2 method on parallel computers. Chem. Phys. Lett..

[52] Dunning TH (1989). Gaussian basis sets for use in correlated molecular calculations. I. The atoms boron through neon and hydrogen. J. Chem. Phys..

[53] Neese F (2012). The ORCA program system: The ORCA program system. Wiley Interdiscip. Rev. Comput. Mol. Sci..

[54] Furche F (2014). Turbomole: Turbomole. Wiley Interdiscip. Rev. Comput. Mol. Sci..

[55] Schirmer J (1982). Beyond the random-phase approximation: A new approximation scheme for the polarization propagator. Phys. Rev. A.

[56] Austin AJ, Frisch MJ, Montgomery JA, Petersson GA (2002). An overlap criterion for selection of core orbitals. Theor. Chem. Acc. Theory Comput. Model. Theor. Chim. Acta.

[57] Ramazanov RR, Maksimov DA, Kononov AI (2015). Noncanonical Stacking Geometries of Nucleobases as a Preferred Target for Solar Radiation. J. Am. Chem. Soc..

[58] Antao VP, Gray DM (1993). CD Spectral Comparisons of the Acid-Induced Structures of Poly[d(A)], Poly[r(A)], Poly[d(C)], and Poly[r(C)]. J. Biomol. Struct. Dyn..

[59] Holm AIS, Nilesen LM, Kohler B, Hoffmann SV, Nielsen SB (2010). Electronic coupling between cytosine bases in DNA single strands and i-motifs revealed from synchrotron radiation circular dichroism experiments. Phys. Chem. Chem. Phys..

[60] Gehring K, Leroy J-L, Guéron M (1993). A tetrameric DNA strucutre with protonated cytosine-cytosine bais pairs. Nature.

[61] Kononov AI, Bakulev VM, Rappoport VL (1993). Exciton effects in dinucleotides and polynucleotides. *J*. Photochem Photobiol B Biol.

[62] Kononov AI, Bukina MN (2002). Luminescence Excitation Spectra Reveal Low-lying Excited States in Stacked Adenine Bases. J. Biomol. Struct. Dyn..

[63] Birks, J. B. *Photophysics of Aromatic**Molecules*. (Wiley‐Interscience, 1970).

[64] Olaso-González G, Merchán M, Serrano-Andrés L (2009). The Role of Adenine Excimers in the Photophysics of Oligonucleotides. J. Am. Chem. Soc..

[65] Serrano-Pérez JJ, González-Ramírez I, Coto PB, Merchán M, Serrano-Andrés L (2008). Theoretical Insight into the Intrinsic Ultrafast Formation of Cyclobutane Pyrimidine Dimers in UV-Irradiated DNA: Thymine versus Cytosine. J. Phys. Chem. B.

[66] Borrego-Varillas R, Cerullo G, Markovitsi D (2019). Exciton Trapping Dynamics in DNA Multimers. J. Phys. Chem. Lett..

