# Supplementary material for: Exciton Absorption and Luminescence in i-Motif DNA
Source: Sci Rep. 2019 Nov 5;9:15988. doi: 10.1038/s41598-019-52242-1 (PMC6831829; doi:10.1038/s41598-019-52242-1)
Supplement: Supplementary file 1 — Supplementary figures and tables [file 41598_2019_52242_MOESM1_ESM.pdf]

Supporting information

Exciton Absorption and Luminescence in i-Motif DNA

Zakhar V. Reveguk[†], *Evgeny V. Khoroshilov*[§], *Andrey. V. Sharkov*[§], *Vladimir A. Pomogaev*[‖ #], *Andrey A. Buglak*[†], *Alexander N. Tarnovsky*[‡] *and Alexei I. Kononov*[†,*]

[†] Department of Molecular Biophysics and Polymer Physics, Saint-Petersburg State University, 199034 St. Petersburg, Russia

[§] P.N. Lebedev Physical Institute, Russian Academy of Sciences, 53 Leninsky Pr., 119991 Moscow, Russia

‖ Department of Physics, Tomsk State University, Tomsk 634050, Russia

Department of Chemistry and Green-Nano Materials Research Center, College of Natural Sciences, Kyungpook National University 1370 Sankyuk-dong, Buk-gu, Daegu 702-701, Republic of Korea

[‡] Department of Chemistry and the Center for Photochemical Sciences, Bowling Green State University, Bowling Green, OH, USA

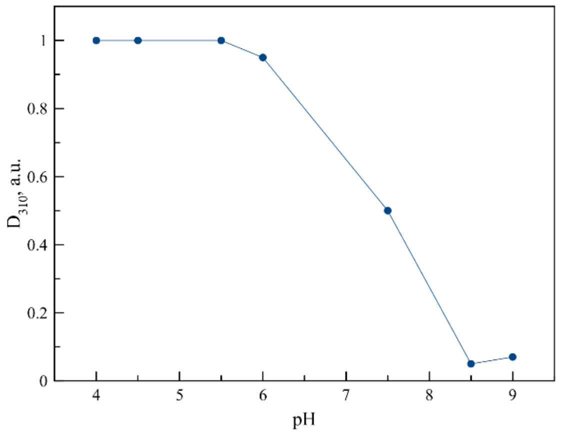

Figure S1. UV titration curve for $(dC)_{10}$, λ=310 nm.

Table S1. Energies of excited electronic states (vertical transition energies, given in eV) of cytosine (Cyt), 1-methylcytosine (1-MeCyt), and cytosine with the frozen core approximation (Cyt, fr) in vacuum.

States \ System	1-MeCyt	Cyt	Cyt, fr
S_1 (ππ*)	4.39	4.46	4.46
S_2 (*n*π*)	4.68	4.69	4.69
S_3 (*n*π*)	5.12	5.14	5.14
S_4 (ππ*)	5.29	5.39	5.39

Table S2. Energies of vertical excitations (eV) with oscillator strengths (in parentheses) of neutral (Cyt) and protonated ($CytH^+$) cytosine as well as their double protonated i-motif tetramer complex (Cyt_42H^+) in water continuum solvent model COSMO. Charge difference densities (CDD)[1] for electronic transitions of Cyt_42H^+, where dark and light colors stand for hole and electron, respectively.

state (type)	CDD \ System	Cyt_42H^+	Cyt	$CytH^+$
S_1 (ππ*)	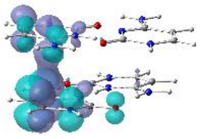	4.27 (0.012)		

S_2 ($\pi\pi$*)	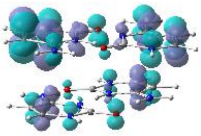	4.48 (0.008)		
S_3 ($\pi\pi$*)	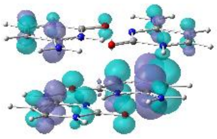	4.56 (0.422)		4.59 (0.265)
S_4 ($\pi\pi$*)	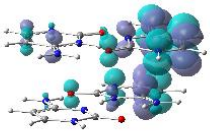	4.64 (0.051)	4.75 (0.126)	
S_5 ($\pi\pi$*)	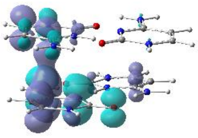	4.93 (0.239)	5.40(0.224)	
S_6 ($\pi\pi$*)	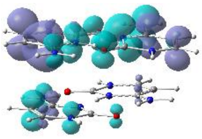	5.09 (0.051)		
S_7 ($\pi\pi$*)		5.19 (0.007)		
S_8 ($\pi\pi$*)		5.24 (0.007)		
S_9 ($\pi\pi$*)		5.32 (0.010)		
S_{10} (nπ*)		5.37 (0.013)		
S_{11} (nπ*)		5.42 (0.026)		
S_{12} ($\pi\pi$*)		5.44 (0.015)		
$\pi\pi^*$				5.95 (0.120)
$n\pi^*$			5.51 (0.003)	6.10 (0.000)

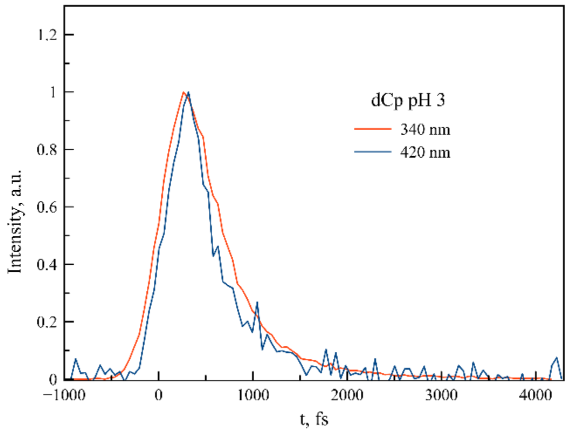

Figure S2. Fluorescence decay curves for dCp at pH 3 recorded at 340 and 420 nm.

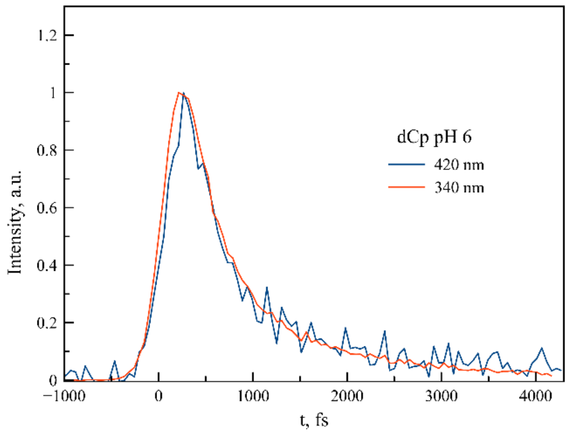

Figure S3. Fluorescence decay curves for dCp at pH 6 recorded at 340 and 420 nm.

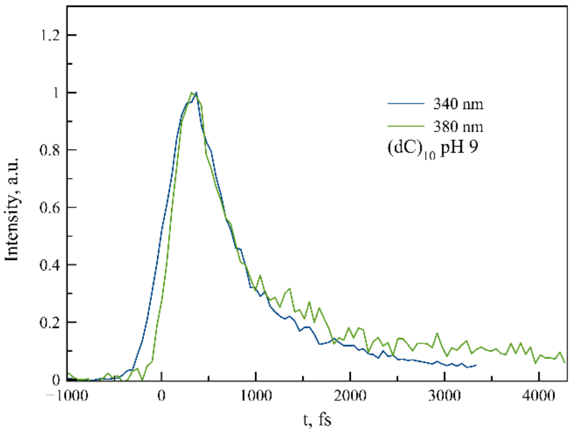

Figure S4. Fluorescence decay curves for $(dC)_{10}$ at pH 9 recorded at 340 and 380 nm.

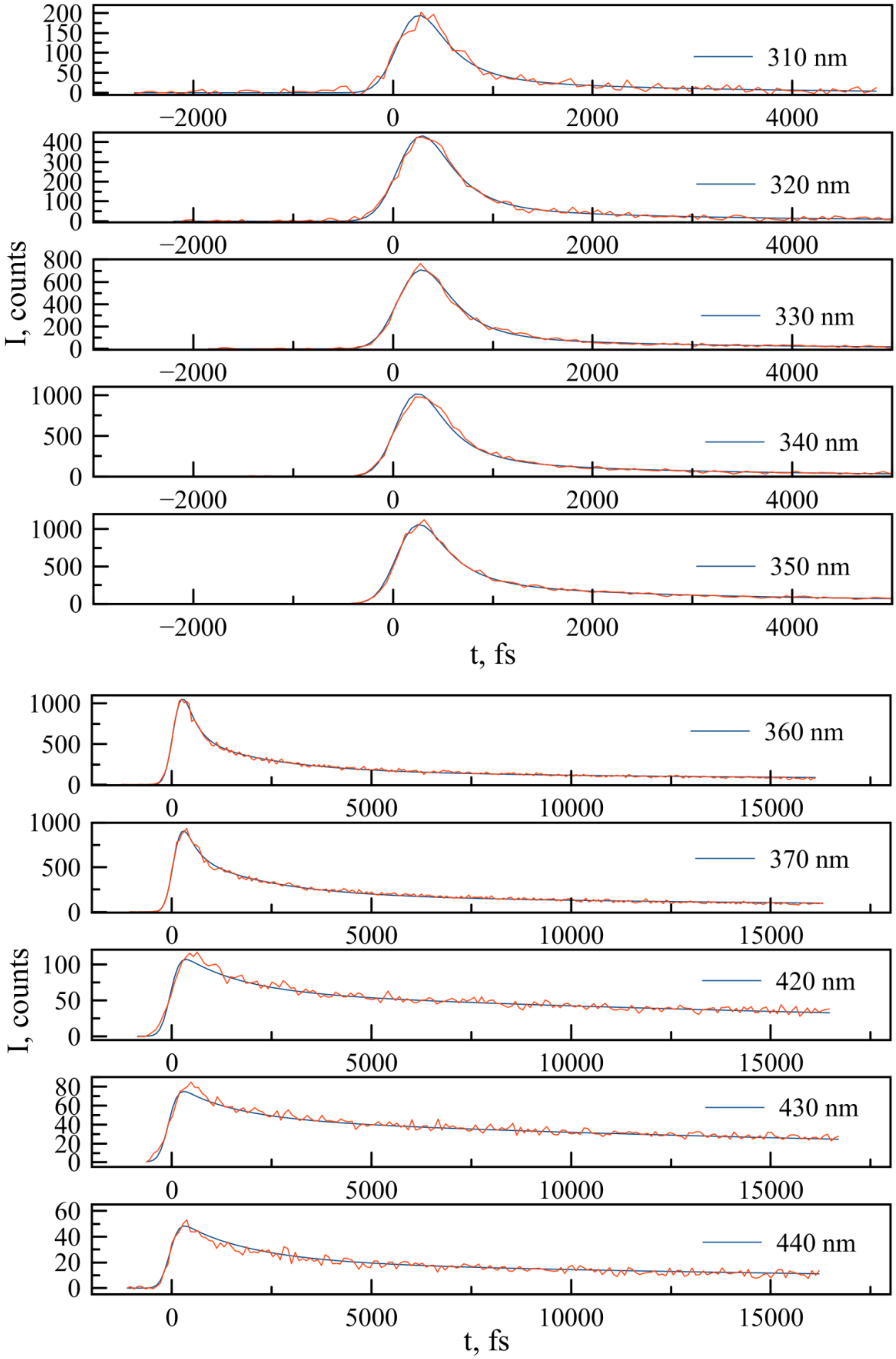
310 nm
320 nm
330 nm
340 nm
350 nm
I, counts
t, fs
360 nm
370 nm
420 nm
430 nm
440 nm
I, counts
t, fs

Figure. S5. Fluorescence decay curves and fits for $(dC)_{10}$ in water (pH 6) at different wavelengths.

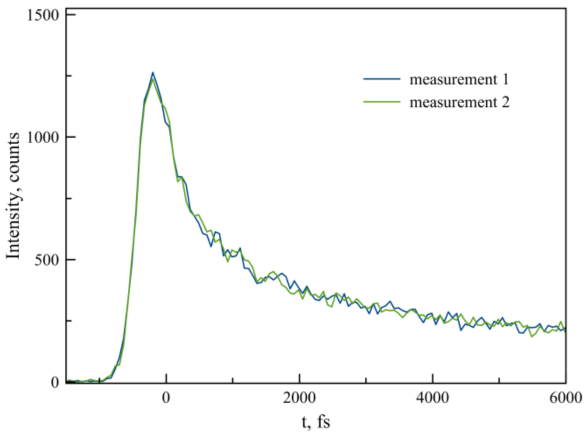

Figure S6. Fluorescence decay curves for $(dC)_{10}$ at pH 6 recorded at 360 nm in two back-to-back measurements.

REFERENCES

1. Beenken, W. J. D. & Pullerits, T. Spectroscopic Units in Conjugated Polymers: A Quantum Chemically Founded Concept? *J. Phys. Chem. B* **108**, 6164-6169 (2004).
